# A Fully Self‐Powered Wearable Leg Movement Sensing System for Human Health Monitoring

**DOI:** 10.1002/advs.202303114

**Published:** 2023-08-17

**Authors:** Jinfeng Yuan, Yuzhong Zhang, Caise Wei, Rong Zhu

**Affiliations:** ^1^ State Key Laboratory of Precision Measurement Technology and Instruments Department of Precision Instrument Tsinghua University Beijing 100084 China

**Keywords:** edge computing, motion monitoring, self‐powered, stretchable strain sensor, thermoelectric generators

## Abstract

Energy‐autonomous wearable human activity monitoring is imperative for daily healthcare, benefiting from long‐term sustainable uses. Herein, a fully self‐powered wearable system, enabling real‐time monitoring and assessments of human multimodal health parameters including knee joint movement, metabolic energy, locomotion speed, and skin temperature, which are fully self‐powered by highly‐efficient flexible thermoelectric generators (f‐TEGs) is proposed and developed. The wearable system is composed of f‐TEGs, fabric strain sensors, ultra‐low‐power edge computing, and Bluetooth. The f‐TEGs worn on the leg not only harvest energy from body heat and supply power sustainably for the whole monitoring system, but also serve as zero‐power motion sensors to detect limb movement and skin temperature. The fabric strain sensor made by printing PEDOT: PSS on pre‐stretched nylon fiber‐wrapped rubber band enables high‐fidelity and ultralow‐power measurements on highly‐dynamic knee movements. Edge computing is elaborately designed to estimate multimodal health parameters including time‐varying metabolic energy in real‐time, which are wirelessly transmitted via Bluetooth. The whole monitoring system is operated automatically and intelligently, works sustainably in both static and dynamic states, and is fully self‐powered by the f‐TEGs.

## Graphical abstract

A fully self‐powered wearable system is developed for monitoring knee joint movement, skin temperature, locomotion speed, and metabolic energy, which is entirely powered by highly‐efficient flexible thermoelectric generators (f‐TEGs). The wearable system integrates f‐TEGs, fabric strain sensor, edge computing, and Bluetooth, which works sustainably in both static and dynamic states, enabling real‐time monitoring and assessment of human motion and health.

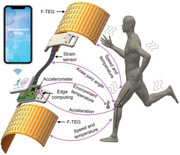

## 1 Introduction

In the current era of the intelligent Internet of Things, wearable electronics are experiencing explosive growth,^[^ ^1^ ^]^ being convenient for various applications of healthcare,^[^ ^2^ ^]^ disease diagnosis/treatment,^[^ ^3^ ^]^ rehabilitation,^[^ ^4^ ^]^ virtual reality,^[^ ^5^ ^]^ human‐machine interaction, and sports training.^[^ ^6^ ^]^ The power supply of wearable devices is an important issue and involves near‐field transmission,^[^ ^7^ ^]^ nuclear,^[^ ^8^ ^]^ battery, and self‐power.^[^ ^9^ ^]^ Among them, self‐powered electronics are considered to be a promising way because of their long‐term sustainability, renewability, safety, and environmental friendliness. However, continuously monitoring health parameters with a fully self‐powered wearable system still faces formidable challenges.

In terms of energy supply, existing wearable devices have tried to translate the common battery‐dependent mode into a self‐powered mode.^[^ ^10^ ^]^ Two ways are mainly adopted, one is passive power supply sensing mode (PPSS), and the other is active power generation sensing mode (APGS). The PPSS mode refers to the use of additional energy harvesting devices to power sensors. Commonly used energy harvesting principles include piezoelectric,^[^ ^11^ ^]^ triboelectricity,^[^ ^12^ ^]^ thermoelectric,^[^ ^13^ ^]^ electromagnetic,^[^ ^14^ ^]^ photoelectricity,^[^ ^15^ ^]^ biofuels,^[^ ^16^ ^]^ electrets,^[^ ^17^ ^]^ and so on. The APGS mode means that the generators work simultaneously as sensing elements. For instance, a triboelectric nanogenerator senses motion.^[^ ^18^ ^]^ The power supply for wearable electronics can also be divided into a sensor‐only power supply and an overall system power supply. The vast majority of self‐powered electronics belong to sensor‐only power supplies.^[^ ^19^ ^]^ Its deficiency is obvious that the back‐end system still needs a battery, and long‐term sustainable uses cannot be achieved. The overall system power supply requires the whole wearable device or system to be fully self‐powered by an onboard generator. However, since the state‐of‐the‐art micro/nano energy harvesting technology is still limited by low energy conversion efficiency, fully self‐powered wearable monitoring is facing a big difficulty. In particular, a fully self‐powered sensing system for sustainable monitoring is a great challenge. Most energy harvesting devices work conditionally. For example, triboelectric generators and piezoelectric generators fail to work in static states, photoelectric generators are invalid in the dark, and biofuel generators require human sweat as fuel and have a limited lifespan. Comparatively, thermoelectric energy harvesting exhibits advantages of sustainability, green, and DC power output.

In terms of intelligent perception, with the vigorous development of artificial intelligence, popular wearable devices have shown the development trend of intellectualized sensing incorporated with machine learning and edge computing, for example, in‐sensor machine learning for full‐body avatar reconstruction.^[^ ^20^ ^]^ It is foreseeable that wearable electronics would be developed into an intelligent agent parasitic on the human body, which puts forward requirements on multimodal perception, onboard data processing, and interaction, as well as wireless transmission.^[^ ^21^ ^]^ This demand also poses a big difficulty to self‐powered devices. However, as public expectations of advanced daily healthcare, fully self‐powered wearable electronics with the ability of intellectualized multimodal sensing and data fusion/transmission are imperative but challenging.^[^ ^22^ ^]^

As mentioned above, thermoelectric energy harvesting has the advantages of sustainability and green. Utilizing thermoelectric harvesting, human body heat can serve potentially as an ideal and sustainable bioenergy source for powering wearable electronics. Besides, body/skin temperature contains abundant information that reflects human health function, such as intestinal flora and metabolite level.^[^ ^23^ ^]^ Long‐term monitoring of skin temperature contributes to preventing diseases such as allergy,^[^ ^24^ ^]^ gout, and arthritis.^[^ ^25^ ^]^ Skin temperature and other physiological parameters related to human activity can quantitatively characterize personal health functions, which are worth to be monitored via wearable electronics, such as stretchable joint sensors.^[^ ^26^ ^]^

Herein, a fully self‐powered limb motion monitoring system is developed for monitoring knee joint movement, skin temperature, locomotion speed, and metabolic energy. The wearable system integrates custom‐made flexible thermoelectric generators (f‐TEG) worn on the thigh and shank for highly‐efficient energy harvesting from body heat, a high‐fidelity and ultra‐low‐power stretchable fabric strain sensor worn on the knee for detecting joint movement, and a commercial accelerometer for detecting limb acceleration, as well as ultra‐low‐power edge computing module (ECM) and Bluetooth. We also propose an f‐TEG‐based zero‐power sensing principle for perceiving skin temperature and locomotion speed. The f‐TEGs not only work as the power generator, but also simultaneously perceive skin temperature and locomotion speed based on a custom‐designed analytic algorithm that achieves ultralow‐power cognitive computing through ECM. Furthermore, a high‐fidelity and ultralow‐power fabric strain sensor is custom‐made by printing PEDOT: PSS ink on a pre‐stretched elastic fabric (nylon fiber‐wrapped rubber band), which captures knee joint movements with a wide sensing range, high fidelity, low hysteresis, and good long‐term reliability. Multimodal health parameters including metabolic energy are figured out in real‐time through the ECM and wirelessly transmitted via Bluetooth. The whole monitoring system is operated automatically and intelligently, works sustainably in both static and dynamic states, and is fully self‐powered by the f‐TEGs.

## 2 Results

### 2.1 Overall Configuration of the Self‐Powered Wearable System

**Figure 1:**
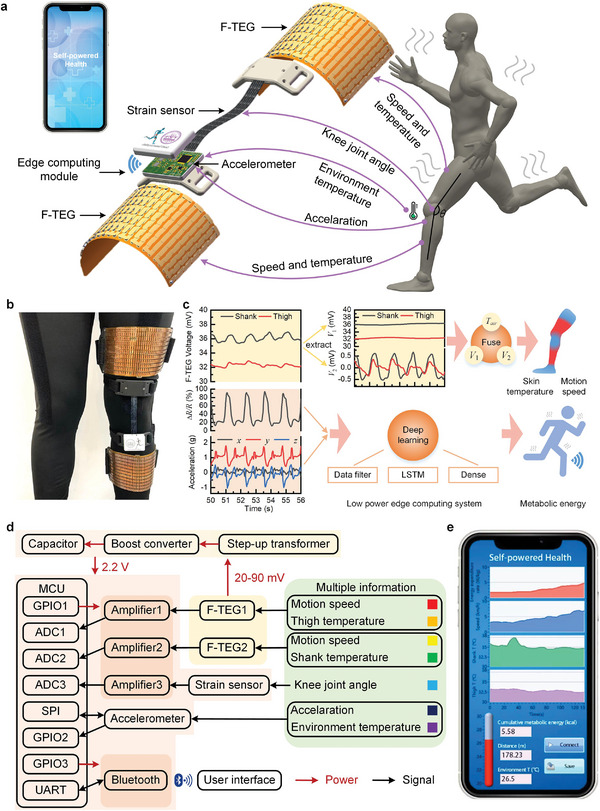
Schematics and images of the self‐powered wearable system. a) 3D structure of the wearable system that enables comprehensive motion monitoring through a synergistic fusion of f‐TEGs, a strain sensor, and an accelerometer. b) Prototype photograph of the wearable system worn on a subject. c) Edge computing framework for estimating skin temperature, locomotion speed, and metabolic energy expenditure. d) Signal flow diagram of the electronic system of the wearable device. MCU, Microcontroller unit. The red arrow indicates the power supply, and the black arrow indicates the signal transmission. e) Custom‐made mobile application for locomotion speed, skin temperature, environment temperature, and metabolic energy tracking.

The wearable system consists of two f‐TEGs, a stretchable strain sensor, an accelerometer, an ECM, and a wireless Bluetooth (**Figure** **1**a). The system can be conformally attached to the leg in virtue of its flexible design (Figure 1b). Two f‐TEGs are made into bandages and worn on the wearer's thigh and shank respectively. Taking advantage of the natural temperature difference between the human body and the environment, the f‐TEGs provide continuous and green energy for the wearable system. Simultaneously, the f‐TEGs also sense the skin temperature and locomotion speed, and thus serve as zero‐power motion sensors.

The stretchable strain sensor worn on the knee monitors the knee joint movement. The accelerometer worn on the shank detects limb acceleration and environment temperature. The ECM is comprised of a power management module, sensor signal conditioning module, and ultra‐low power microcontroller unit (MCU), integrated with a Bluetooth module. The ECM takes responsibility to make data fusion of the detected multimodal sensing parameters for assessing human health function.

Concretely, the DC component (*V*_1_) and AC component (*V*_2_) of the f‐TEG output are utilized to extract the locomotion speed and skin temperature during human walking and running. The real‐time metabolism energy can be estimated using the strain sensor signal (Δ*R/R*) and the accelerometer‐detected acceleration through a data‐driven model based on deep learning (Figure 1c). The block diagram illustrates the energy flow and signal flow of the electronic system (Figure 1d). In the energy flow, the f‐TEG produces a voltage ranging from 20 to 90 mV through thermoelectric conversion. This voltage is boosted to 2.2 V using a DC‐DC converter (LTC3108‐1) to power the entire electronic system. A smart power management scheme is designed, that is the MCU and the accelerometer always power on, while the strain sensor and Bluetooth always power off until a human activity is detected. In the signal flow, the output voltage of f‐TEG is amplified through a conditioning circuit composed of the amplifiers, and then converted into digital information through an analog‐to‐digital converter (ADC). A monitoring application is demonstrated to continuously monitor human health parameters by using the self‐powered wearable system (Figure 1e; Movie S1, Supporting Information).

### 2.2 F‐TEG Design and Sensing for Skin Temperature and Locomotion Speed

**Figure 2:**
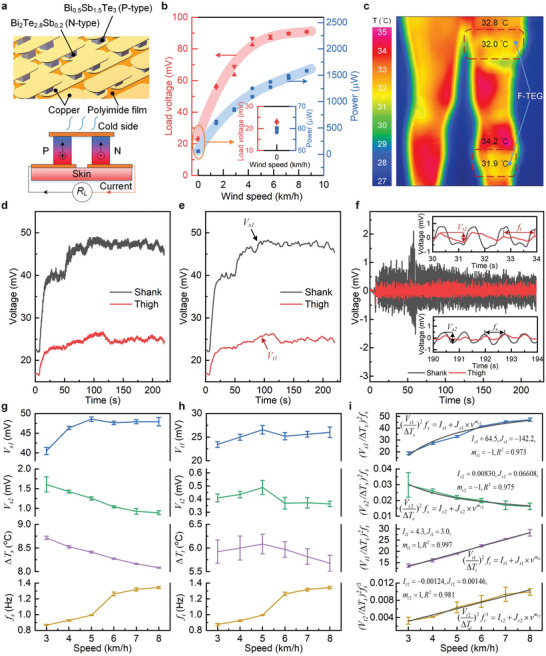
Schematics and characterizations of the f‐TEG used as an energy harvester and motion sensor. a) Magnified view of the structure of the thermoelectric unit. b) Output voltage and power of the f‐TEG under different wind speeds when loaded with a boost converter. c) Infrared thermography of temperature distribution when the f‐TEG is worn on the lower limb. d) The output voltage of the f‐TEG when the walking/running speed of the subject is 3–8 km h^−1^. e) DC component extracted from the output voltage of the f‐TEG. f) AC component extracted from the output voltage of the f‐TEG. g) DC component (*V_s_*_1_) and AC component (*V_s_*_2_) of the f‐TEG worn on the shank, temperature difference (Δ*T_s_*) between shank skin and environment, and gait frequency (*f_s_*) extracted from *V_s_*_2_ at different motion speeds. h) DC component (*V_t_*_1_) and AC component (*V_t_*_2_) of the f‐TEG worn on the thigh, temperature difference (Δ*T_t_*) between thigh skin and environment, and gait frequency (*f_t_*) extracted from *V_t_*_2_ at different motion speeds. i) The model fitting results of formula 5–8. Colorful line: experimental true value; Black line: fitting value.

Based on the Seebeck effect, the f‐TEG is comprised of P‐type (Bi_0.5_Sb_1.5_Te_3_) and N‐type (Bi_2_Te_2.8_Se_0.2_) thermoelectric particles in an array assembled on a flexible polyimide (PI) substrate (**Figure** **2**a). The overall f‐TEG size is 16 × 8 cm^2^. The f‐TEG is optimized to contain 896 thermoelectric particles and a fill factor of 7% by using a systematic optimization method taking multi‐objective optimization of power density, material consumption, and power matching with wearable sensory system.^[^ ^27^ ^]^ The 896 particles are divided into two equal parts in series and then connected in parallel. Details of structure design and fabrication are described in Experimental Section and Figure S1 (Supporting Information).

The fabricated f‐TEG can conformally attach to human limbs, has high durability (Figure S2, Supporting Information) and capably affords long‐term monitoring during walking and running. The f‐TEG is tested and the testing performance is shown in Figure 2b. A wind speed of 0–9 km h^−1^ mimicking the wind flow induced by human walking and running is used in the testing experiment (See Experimental Section for more details). The output voltage of the f‐TEG reaches 20–90 mV under the wind speed of 0–9 km h^−1^, and the output power reaches 60–1600 µW. The f‐TEG achieves highly efficient thermoelectric conversion. Figure 2c shows an infrared thermography of temperature distribution when the f‐TEG is worn on the lower limb. It can be seen that the skin temperature of the shank (34.2 °C) is higher than that of the thigh (32.8 °C). The temperature difference between the hot and cold sides of the f‐TEG worn on the shank is about 2.3 °C, while the temperature difference between the hot and cold sides of the f‐TEG on the thigh is less than 1 °C. Therefore, the energy harvesting on the shank is much more than that on the thigh. It can be also validated by the output voltages of the two f‐TEGs worn on the shank and thigh of a subject who is walking/running at a speed of 3–8 km h^−1^, shown in Figure 2d. The f‐TEG worn on the shank has a higher output voltage, indicating higher energy harvesting.

Besides, the f‐TEG can be also utilized to perceive human motion and skin temperature via a data fusion of its output voltages based on the convective effect on thermoelectric conversion. To minimize the computing energy cost, a mathematical analytical method is proposed to estimate human motion and skin temperature from the f‐TEG sensing using edge computing. We extract the DC component (Figure 2e) and AC component (Figure 2f) from the f‐TEG signals (Figure 2d). The DC component of the shank is defined as *V_s_*_1_, the amplitude of the AC component of the shank is defined as *V_s_*_2_, and the gait frequency of the shank (*f_s_*) is extracted from the AC component. The temperature difference between the shank skin temperature and environment temperature is defined as Δ*T_s_*.

Figure 2g shows the detected parameters *V_s_*_1_, *V_s_*_2_, Δ*T_s_*, and *f_s_* varying with the locomotion speed (*v*). Correspondingly, the parameters of the thigh are characterized as *V_t_*_1_, *V_t_*_2_, Δ*T_t_*, and *f_t_* respectively, and the results are shown in Figure 2h. Parameter definitions are also described in Table S1 (Supporting Information).

Generally, according to the principle of convective heat transfer, the relationship between the output voltage (*U*) of f‐TEG and the motion‐induced wind speed (*v*) can be characterized by the King formula:^[^ ^28^ ^]^

(1) $$U^{2}=I+J\times v^{m}$$

where *I*, *J*, and *m* are the coefficients.

The output of f‐TEG responds to not only the motion speed, but also the temperature difference (Δ*T*) between the skin and the environment. We assume a simplified relationship between the f‐TEG output and the temperature difference Δ*T*, and substitute it into Equation (1):

(2) $$U=V_{i}/\Delta T$$

(3) $${\left(V_{i}/\Delta T\right)}^{2}=I+J\times v^{m}$$

where *V_i_* represents the AC or DC component of the f‐TEG output.

Furthermore, taking the gait frequency into account, the above equation is modified as:

(4) $${\left(V_{i}/\Delta T\right)}^{2}\times f^{n}=I+J\times v^{m}$$

where *f* represents the gait frequency, and *n* is the fitting coefficient. To simplify edge computing, *n* and *m* are set as integers. The coefficients of *I*, *J*, *m*, and *n* are determined by a data‐driven model fitting. Specific model optimization is described in Figures S3 and S4 (Supporting Information).

After the optimization, the mathematical analytic models for estimating human locomotion speed and skin temperatures from the f‐TEG sensing are determined as follows:

(5) $${\left(V_{s1}/\Delta T_{s}\right)}^{2}\times f_{s}=I_{s1}+J_{s1}\times v^{m_{s1}}$$

(6) $${\left(V_{s2}/\Delta T_{s}\right)}^{2}\times f_{s}=I_{s2}+J_{s2}\times v^{m_{s2}}$$

(7) $${\left(V_{t1}/\Delta T_{t}\right)}^{2}\times f_{t}=I_{t1}+J_{t1}\times v^{m_{t1}}$$

(8) $${\left(V_{t2}/\Delta T_{t}\right)}^{2}\times f_{t}^{3}=I_{t2}+J_{t2}\times v^{m_{t2}}$$

(9) $$\Delta T_{s}=T_{s}-T_{air}$$

(10) $$\Delta T_{t}=T_{t}-T_{air}$$

where *T_s_*, *T_t_*, and *T_air_* represent the skin temperature of the shank, the skin temperature of the thigh, and the environment temperature, respectively. *I_s_*_1_, *J_s_*_1_, *m_s_*_1_, *I_s_*_2_, *J_s_*_2_, *m_s_*_2_, *I_t_*_1_, *J_t_*_1_, *m_t_*_1_, *I_t_*_2_, *J_t_*_2_, and *m_t_*_2_ are the fitting coefficients, and the model fitting results are shown in Figure 2i. The model fitting results indicate that the established mathematical models agree very well with the experimental data. Therefore, the parameters of the locomotion speed *v*, the skin temperatures of shank and thigh (*T_s_*, *T_t_*) can be figured out by combining the Equations (5)–(10) according to the AC and DC components of the f‐TEG outputs. Details can be seen in Note S1 (Supporting Information). This analytic algorithm for estimating human locomotion speed and skin temperature from f‐TEG sensing can greatly reduce the computational complexity and thus minimizes computing energy cost.

### 2.3 Design of the Stretchable Strain Sensor

High fidelity in monitoring highly‐dynamic knee joint movements during human walking and running is very important for diagnoses of abnormal gait and arthritis, as well as assessing motor rehabilitation training. However, most strain sensors using conductive elastomers suffer from signal distortions in monitoring highly‐dynamic joint movements.^[^ ^6c^ ^]^ The reason is that incongruous dynamic behaviors of conductive pathways along transverse and lengthways directions in conductive active materials generally exist and distort the electric responses of conductive elastomers to dynamic strains.^[^ ^6c^ ^]^ To overcome this signal distortion issue, a structural design on the conductive elastomer is significant.

**Figure 3:**
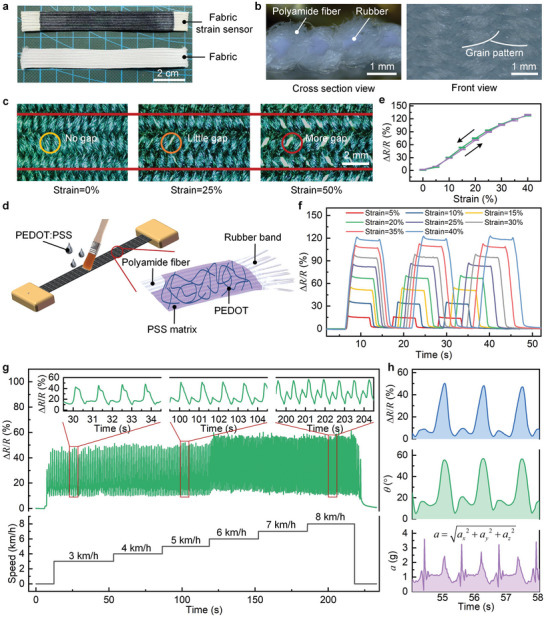
Preparation and characterizations of the fabric/PEDOT: PSS stretchable strain sensor used for knee joint angle monitoring. a) The fabrics before and after printing PEDOT: PSS ink. b) Cross section and front view of the elastic bandage fabric. c) Deformation of the fabric strain sensor under different strains. d) Printing PEDOT: PSS solution on polyamide fabric and dry to prepare the strain sensor. e) Relative resistance changes and hysteresis characteristics of the strain sensor under different strains. f) Resistance responses during three stretch‐release cycles under different strains. g) Resistance responses of the strain sensor worn on the knee under different locomotion speeds. h) Fidelity comparison between our strain sensor signal (ΔR/R) and accelerometer signal (a) when monitoring knee joint angle (θ). The ground‐truth knee joint angle (θ) is detected using standard equipment.^[^ ^29^ ^]^

We propose and develop a fabric/PEDOT: PSS stretchable strain sensor by printing an organic conductor of PEDOT: PSS on an elastic bandage fabric (**Figure** **3**a). Compared with other flexible substrates, the elastic bandage fabric takes advantage of low cost, good air permeability, and comfortable wearing. The fabric is custom‐made of polyamide fiber‐wrapped rubber band. The polyamide fiber bundles are woven around the rubber bands in an “8” shaped orbit (Figure 3b: Cross section view), and the belt body pattern is in a herringbone shape (Figure 3b: Front view). This polyamide fiber‐wrapped rubber band capably suppresses the incongruous conductive behavior. Specifically, the fabric deforms little in the transverse direction during longitudinal stretching, thereby achieving high‐fidelity measurements on dynamic strains. Figure 3c shows the deformation of a fabric strain sensor under different tensile strains. An unstretched polyamide fiber bundles are in close contact. When the fabric is stretched to 25%, visible separation occurs between the polyamide fiber bundles, and a small amount of gap appears in the stretching direction. When it is stretched to 50%, a large amount of gap appears. During this process, the transverse deformation of the fabric is little which guarantees the congruous conductive pathways of the strain sensing, leading to high‐fidelity measurements on dynamic strains.

The organic material PEDOT: PSS is selected as the conductive filler because of its mechanical flexibility, good conductivity, and environmental stability. The stretchable strain sensor is fabricated by printing PEDOT: PSS ink on a pre‐stretched (about 125%) elastic bandage fabric and hot air curing (70 °C) as shown in Figure 3d. The pre‐stretched process allows for better ink infiltration. The resistance value of the fabric strain sensor is customized to be ≈15 kΩ by repeatedly brushing the fabric with PEDOT: PSS ink to realize the ultra‐low energy (≈48 nW) powered directly by the f‐TEG. Figure 3e shows the relative resistance change and hysteresis characteristics of the fabric strain sensor under different strains. The strain sensing range reaches 40% and has good linearity, with a low hysteresis error of 2.8%. Figure 3f shows the responses of the strain sensor in three stretch‐release cycles under different strains, indicating good signal fidelity in monitoring highly‐dynamic stains. The fatigue resistance of the sensor is tested to validate its good long‐term stability, the results during 10 000 reciprocating stretching/releasing cycles are shown in Figure S5 (Supporting Information). The results indicate that the fabric/PEDOT: PSS strain sensor is endowed with good stability and reliability during long‐term uses.

Figure 3g shows the monitoring results of knee joint angle using the fabric/PEDOT: PSS strain sensor during the subject's walking and running at an increasing speed of 3 to 8 km h^−1^. We compare the joint movement result of the strain sensor with that of the accelerometer in Figure 3h. It is seen that the result (Δ*R*/*R*) of the strain sensor is in accord with the ground‐truth knee joint angle (*θ*), while the accelerometer‐detected signal (*a*) is seriously distorted. The peak value of the normalized cross‐correlation coefficient between the sensor signal and the actual joint movement is calculated as the fidelity index, as shown in Figure S6 (Supporting Information). It can be seen that the fidelity of the proposed fabric strain sensor reaches 97%, while the fidelity of the accelerometer is only 84%. The proposed fabric/PEDOT: PSS strain sensor capably monitors the joint movement with high fidelity and low power, which is beneficial to the diagnoses of abnormal gait and arthritis, and assessing motor rehabilitation training.

### 2.4 Real‐Time Metabolic Energy Estimate and Power Management by Edge Computing

**Figure 4:**
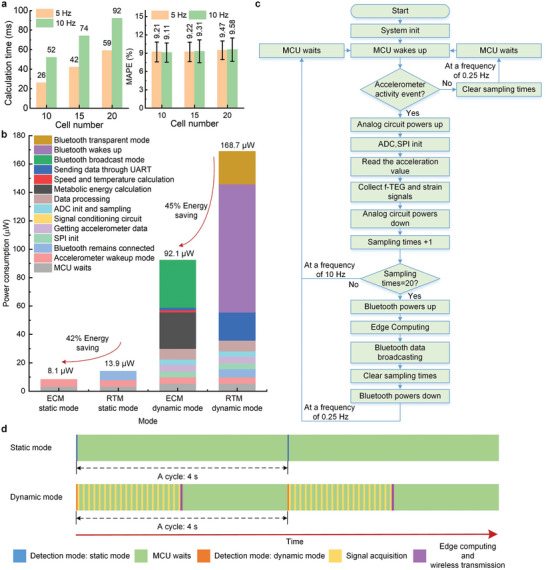
Operation of edge computing in the self‐powered wearable monitoring system. a) The calculating time and accuracy of the deep learning model with different neuron numbers and sampling rates for the metabolism estimation. b) Power consumption of every component in the wearable system using edge computing module (ECM) and real‐time transmission module (RTM), respectively. Static mode indicates that the human body is in a static state. Dynamic mode indicates that the human body is in motion. c) Ultra‐low power management strategy of the wearable system using the ECM. d) Timeline display of the workflow of ECM.

Metabolic energy expenditure reflects the intensity of physical daily activities, which has become one of the key indexes for health assessments.^[^ ^30^ ^]^ In our system, the metabolic energy is estimated in real‐time by fusing the detected motion parameters using an edge computing module (ECM). We chose a long short‐term memory (LSTM) neural network with a single layer to perform this task.^[^ ^31^ ^]^ The input parameters of the neural network are the knee joint movement detected by the strain sensor and the tri‐axial acceleration of the shank detected by the accelerometer, and the metabolic rate to be estimated is the output of the network (More details in Experimental Section). Fewer neurons and shorter input sequence lengths stand for fewer spatiotemporal resources, and thus reduce the computing power consumption of the ECM. The time window length of the dataset is optimized to be 2 s. As shown in **Figure** **4**a, when the cell number of LSTM is within 5–20 and the number of sampling data points is either 10 or 20, the mean absolute percentage error (MAPE) of the model holds at 9%−10%. Therefore, we set the cell number as 5 and the sampling rate as 5 Hz to gain an appropriate estimate accuracy while reducing power consumption. It is tested that the ECM takes 26 ms to estimate the metabolic energy using the established LSTM model, and only 1.4 ms to synchronously figure out the locomotion speed and skin temperature using the derived analytical models of Equations (5)–(10).

Figure 4b shows the power consumption of components in the wearable system. We also compare the power consumption using the ECM to that of the real‐time transmission module (RTM). ECM and RTM are two data processing approaches for the wearable monitoring system. The ECM parses a large amount of sensor data into the physiological parameters using the onboard MCU, and then wirelessly transmits the health parameters to an external terminal. The RTM wirelessly transmits the massive sensor data to an external terminal and makes the data processing in the external terminal. Detailed data can be seen in Table S2 (Supporting Information). The comparisons indicate that the power consumption of ECM is significantly lower than that of RTM in both static and dynamic modes. Static mode means that the human body is in a quiescent state. Dynamic mode means that the human body is in an active state. In static mode, the power consumption of ECM is 8.1 µW, while the power consumption of RTM is 13.9 µW. In dynamic mode, the power consumption of ECM is 92.1 µW, while the power consumption of RTM is 168.7 µW. The ECM gains more than 40% energy savings by avoiding frequent Bluetooth connection and data transmission. As shown in Figure 2b, the output power of the f‐TEG is ≈60 µW in static mode, and exceeds 300 µW in dynamic mode. A DC‐DC converter (LTC3108‐1) is used to boost the output voltage (20–90 mV) of the f‐TEG to 2.2 V. The conversion efficiency of the DC‐DC converter is 35%, and thus the effective output power of the f‐TEG is 21 µW in static mode and exceeds 105 µW in dynamic mode. Comparing the f‐TEG power supply and the power consumption of ECM, it is seen the ECM works sustainably under both static and dynamic conditions.

Figure 4c illustrates the workflow of power management operated by the ECM. The workflow of the RTM is seen in Figure S7 (Supporting Information). The ECM uses the accelerometer signal to recognize whether the human body is active, and the MCU collects the accelerometer signal at a frequency of 0.25 Hz to reduce power consumption. In a quiescent state, the system enters a static mode in that the MCU does not collect the strain and f‐TEG sensing data and stops the parameter estimation and data transmission. Once a human activity is detected, the system enters a dynamic mode in that the MCU collects the strain sensing and f‐TEG sensing data at a sampling rate of 10 Hz. When the collected dataset meets the time length of 2 s, the ECM estimates the locomotion speed, skin temperature, and metabolic energy using the data fusion methods mentioned above and transmits the estimated health parameters to the external terminal through Bluetooth broadcast mode. This parameter estimation and transmission process is operated at a frequency of 0.25 Hz. During the monitoring, the average power consumption of the stretchable strain sensor, the ECM estimating locomotion speed and skin temperature by the analytical model (Equations (5), (6), (7), (8), (9), (10)), and the ECM estimating metabolic energy by the LSTM model is 48 nW, 1.5 µW, and 25.6 µW, respectively. The MCU works continuously in both motion and quiescent states to intelligently manage the working mode and the f‐TEG works sustainably to supply the power for the system. This smart power management capably sustains the monitoring system working continuously. Visually display of this workflow in the form of a timeline can be seen in Figure 4d.

### 2.5 Self‐Powered Wearable Human Motion Monitoring

**Figure 5:**
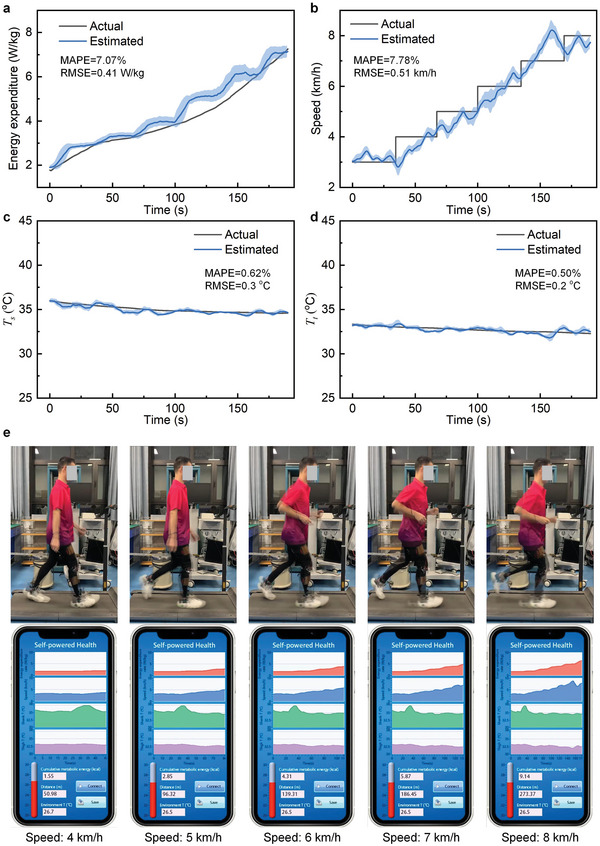
Verification of multimodal health parameter monitoring using the self‐powered wearable system. a) Real‐time metabolic energy estimation. The actual energy expenditure refers to the ground truth expenditure measured by the respiratory oxygen consumption meter. b) Motion speed estimation. The speed of the treadmill is regarded as the actual speed. c) Shank skin temperature estimation. d) Thigh skin temperature estimation. In (c) and (d), the actual skin temperature is measured using a platinum resistance PT1000. In (a–d), the blue error bars are calculated from the dataset over the time. e) Demonstration of human motion monitoring using the wearable system. A subject walks/runs on a treadmill. A wearable leg motion monitoring device is worn on his leg and performs the motion monitoring in real‐time. An intelligent terminal receives the health parameters through Bluetooth.

To validate the effectiveness of the wearable motion monitoring system, we invite a subject to wear the system and walk/run at an increasing speed on a treadmill. The subject speeds up from 3 to 8 km h^−1^ with an interval of 1 km h^−1^, and every speed lasts 0.5 min. During the walking/running, the wearable system monitors the subject's leg movement in real‐time and estimates the metabolic energy expenditure, the locomotion speed, and the skin temperature online. **Figure** **5**a shows the estimated time‐varying metabolic energy of a subject during his walking/running. The estimated mean absolute percentage error (MAPE) of the metabolic energy reaches 7.07% and the root mean square error (RMSE) reaches 0.41 W kg^−1^. During the experiment, the subject wears a spiroergometry system (METALYZER 3B, CORTEX Biophysik Co., Ltd.) to measure the ground‐truth metabolic energy synchronously. Figure 5b shows the locomotion speed estimation of the subject during the walking/running experiment. The MAPE reaches 7.78% and the RMSE reaches 0.51 km h^−1^. The leg skin temperature is also monitored synchronously and the result is shown in Figure 5c (shank) and Figure 5d (thigh).

Finally, we demonstrate an application task, in which a subject wears the fully self‐powered wearable monitoring system on his leg and walks/runs on a treadmill. The self‐powered monitoring system detects the subject's leg movement, estimates health parameters, and wirelessly transmits the parameters to a mobile terminal (e.g., cell phone) at a frequency of 0.25 Hz, which is entirely powered by human body heat. The real‐time metabolic energy, accumulated calorie consumption, locomotion speed, walking/running distance, thigh skin temperature, shank skin temperature, and ambient temperature are monitored continuously and simultaneously as shown in Figure 5e and Movie S1 (Supporting Information).

## 3 Conclusions

In this study, a fully self‐powered leg movement sensing system integrating f‐TEGs, low‐power sensing, edge‐computing, wireless transmission, and smart power management is developed for human daily health monitoring. The f‐TEG achieves highly‐efficient thermoelectric conversion (up to 1600 µW), and exhibits good flexibility and excellent sustainability in terms of wearable thermoelectric performance, allowing a full power supply for the whole monitoring system. Besides, the f‐TEG worn on the leg capably perceives the locomotion speed and the skin temperature using an analytical algorithm. The analytical algorithm enables edge computing to spend only 1.4 ms to figure out the locomotion speed and skin temperature from the f‐TEG sensing, thereby achieving ultra‐low‐power (1.5 µW) cognitive computing. The fabric/PEDOT: PSS stretchable strain sensor contributes to detecting highly‐dynamic knee joint movement with high fidelity (97%), wide sensing range (up to 40%), low hysteresis (2.8%), good long‐term reliability (up to 10 000 cycles), and ultra‐low power (48 nW). Furthermore, human metabolic energy is estimated in real‐time from the detected motion parameters by using onboard edge computing. By elaborately laying out the power management, the wearable system can continuously monitor human motion, and estimate and transmit the health parameters to a terminal device wirelessly, all of which are entirely powered by human body heat. For the first time, this study verifies combining wearable sensing electronics, self‐powered devices, artificial intelligence, and wireless transmission for the application of highly energy‐autonomous intelligent healthcare wear, providing an effective solution to sustainable daily health monitoring.

## 4 Experimental Section

### Materials

The bottom polyimide (PI) substrate (Pyralux AP8525R, 12.5 µm thickness) of the f‐TEG was bought from DuPont Co. Ltd. P‐type (GXSY710) and N‐type (GXSY110) thermoelectric particles are bought from WINGOO Co. Ltd, China. PEDOT: PSS solution (Orgacon DRY, 1.1–1.3 wt.%) is bought from Agfa Materials.

### Fabrication of the f‐TEG

First, the PI copper‐coated film was patterned by a lift‐off process to form the bottom electrodes. Thermoelectric particles (1 × 1 × 3 mm^3^) were bonded onto the bottom electrodes in an array using solder as an adhesive at 200 °C for 5 min. Second, the PI tape (serve as a sacrificial layer) with copper film (100 µm thick) is patterned by a lift‐off process to form the upper electrode, then bond it onto the particles using solder at 200 °C for 5 min to form an inflexible generator. Finally, the inflexible generator was soaked in acetone for 12 h to peel off the PI tape, then the flexible thermoelectric generator was prepared. The fabrication process is seen in Figure S1 (Supporting Information).

### Characterization of the f‐TEG

The f‐TEG adheres to a hot plate and the temperature was set as 34 °C, which was designed to mimic real human skin. The air temperature was 25.5 °C. The airflow blowing over the f‐TEG was generated by a common axial fan. An anemometer (AS8336) was responsible for tracking the wind speed, which ranges from 0 to 9 km h^−1^ corresponding to different human motions including rest, walking, and running. A booster chip (LTC3108‐1) was used as the load of f‐TEG. The load voltage and current were measured by a multimeter (FLUKE 117C).

### Characterization of the Stretchable Strain Sensor

The strain sensor was tested using a tensile tester at a deformation rate of 10 mm s^−1^. The size of the sensor samples was 100 mm × 10 mm × 1 mm. The electrical properties are measured by the 4‐point probes method. The voltage was tracked by a 24‐bit dynamic signal acquisition (MPS‐140801).

### Walking/Running Experimental Setup and Model Training

The subject wears the wearable system over his athletic pant and runs on a treadmill at a room temperature of 26 °C. The running speed was 3 km h^−1^, 4 km h^−1^, 5 km h^−1^, 6 km h^−1^, 7 km h^−1^, and 8 km h^−1^, each speed was maintained for 30 s. The subject runs six times to obtain six datasets. During the experiment, the actual energy metabolism was measured by using a spiroergometry system (METALYZER 3B, CORTEX Biophysik Co., Ltd.).

As for the model training and testing, the leave‐one‐out cross‐validation method was used. Specifically, five datasets were used as training sets, and the remaining one dataset was used as the testing set. Each dataset of six datasets was used as the testing set in turn to eliminate the contingency of the testing model. The training set had 45 000 samples and the test set had 9000 samples.

### Hardware and Software Design of the Wearable Device

A DC‐DC converter (LTC3108‐1) was used to boost the output of f‐TEG to 2.2 V. Ultra‐low power amplifiers (LPV812) were used to build the signal conditioning circuit of the f‐TEG and the strain sensor. A microcontroller unit (STM32L476RGT6) was used to collect signals, and preprocess signals and acts as a hardware carrier of the edge computing module. The basic frequency of the MCU was set to a lower 8 MHz to reduce the operating power consumption. An accelerometer (ADXL362) was used to track the acceleration of the shank. A low‐power Bluetooth module (DA14531) was used for wireless data transmission.

Data processing and calculation were performed on the MATLAB R2022a platform. The training of the deep learning model was performed by Python 3.8 and Tensorflow 2.5. The mobile terminal software was designed by NI LabVIEW2018.

Experiments performed in this study involving human participants were approved by the Institution Review Board of Tsinghua University (No. 20 180 009). And informed consent was obtained from human subjects to use their images and conduct the experiments described in this paper.

## Conflict of Interest

The authors declare no conflict of interest.

## Supporting information

Supporting Information

Supplemental Movie 1

## Data Availability

The data that support the findings of this study are available from the corresponding author upon reasonable request.

## References

[1] a) K. Meng , X. Xiao , W. Wei , G. Chen , A. Nashalian , S. Shen , X. Xiao , J. Chen , Adv. Mater. 2022, 34, 2109357; 10.1002/adma.202109357 35044014

[2] M. Wang , Y. Yang , J. Min , Y. Song , J. Tu , D. Mukasa , C. Ye , C. Xu , N. Heflin , J. S. McCune , T. K. Hsiai , Z. Li , W. Gao , Nat. Biomed. Eng. 2022, 6, 1225. 35970928 10.1038/s41551-022-00916-z PMC10432133

[3] a) S. Han , S. K. Lee , J. W. Kim , S. Bae , S. H. Bae , K. H. Choi , J. S. Kim , Mater. Horiz. 2022, 9, 2846; 36052699 10.1039/d2mh00692h

[4] S. Gao , T. He , Z. Zhang , H. Ao , H. Jiang , C. Lee , Adv. Sci. 2021, 8, 2101834. 10.1002/advs.202101834 PMC8529439 34414697

[5] a) H. Fang , J. Guo , H. Wu , Nano Energy 2022, 96, 107112;

[6] a) X. Dai , L. B. Huang , Z. Sun , Y. Du , B. Xue , M. C. Wong , J. Han , Q. Liang , Y. Wu , B. Dong , J. Kong , J. Hao , Mater. Horiz. 2022, 9, 2603; 35942798 10.1039/d2mh00534d

[7] a) Y. Jia , G. Liu , G. Xu , X. Li , Z. Shi , C. Cheng , D. Xu , Y. Lu , Q. Liu , Sens. Actuator B‐Chem. 2022, 367, 132050;

[8] S. Dhanekar , K. Rangra , Adv. Mater. Technol. 2021, 6, 2000895.

[9] a) D. K. Bharti , S. Veeralingam , S. Badhulika , Mater. Horiz. 2022, 9, 663; 34907407 10.1039/d1mh01606g

[10] a) L. Liang , H. Lv , X. L. Shi , Z. Liu , G. Chen , Z. G. Chen , G. Sun , Mater. Horiz. 2021, 8, 2750; 34617552 10.1039/d1mh00775k

[11] a) Y. Zhang , L. Zhou , C. Liu , X. Gao , Z. Zhou , S. Duan , Q. Deng , L. Song , H. Jiang , L. Yu , S. Guo , H. Zheng , Nano Energy 2022, 99, 107420;

[12] a) Y. Qin , J. Mo , Y. Liu , S. Zhang , J. Wang , Q. Fu , S. Wang , S. Nie , Adv. Funct. Mater. 2022, 32, 2201846;

[13] a) C. Chen , R. Wang , X. L. Li , B. Zhao , H. Wang , Z. Zhou , J. Zhu , J. W. Liu , Nano Lett. 2022, 22, 4131; 35536152 10.1021/acs.nanolett.2c00872

[14] a) T. Bhatta , G. B. Pradhan , K. Shrestha , S. Lee , S. M. S. Rana , S. Sharma , H. Song , S. Jeong , J. Y. Park , Nano Energy 2022, 103, 107860;

[15] a) Y. Guo , Y. Li , Q. Zhang , H. Wang , J. Mater. Chem. C 2017, 5, 1436;

[16] a) M. C. Hartel , D. Lee , P. S. Weiss , J. Wang , J. Kim , Biosens. Bioelectron. 2022, 215, 114565; 35926393 10.1016/j.bios.2022.114565

[17] a) X. Ma , X. Chen , X. Xiang , F. Zhang , Y. Zhao , F. Wang , X. Mu , Y. Dai , P. He , X. Zhang , Nano Energy 2022, 103, 107729;

[18] a) X. Pu , H. Guo , J. Chen , X. Wang , Y. Xi , C. Hu , Z. L. Wang , Sci. Adv. 2017, 3, e1700694; 28782029 10.1126/sciadv.1700694 PMC5533541

[19] a) L. Wang , Y. Tang , Y. Li , C. Liu , N. Wei , W. Zeng , D. Liang , ACS Appl Mater Interfaces 2022, 14, 47136; 36200953 10.1021/acsami.2c15117

[20] H. Yang , J. Li , X. Xiao , J. Wang , Y. Li , K. Li , Z. Li , H. Yang , Q. Wang , J. Yang , J. S. Ho , P. L. Yeh , K. Mouthaan , X. Wang , S. Shah , P. Y. Chen , Nat. Commun. 2022, 13, 5311. 36085341 10.1038/s41467-022-33021-5 PMC9461448

[21] A. Alagumalai , W. Shou , O. Mahian , M. Aghbashlo , M. Tabatabaei , S. Wongwises , Y. Liu , J. Zhan , A. Torralba , J. Chen , Z. Wang , W. Matusik , Joule 2022, 6, 1475.

[22] a) Y. Gai , E. Wang , M. Liu , L. Xie , Y. Bai , Y. Yang , J. Xue , X. Qu , Y. Xi , L. Li , D. Luo , Z. Li , Small Methods 2022, 6, 2200653; 10.1002/smtd.202200653 36074976

[23] a) L. E. Armstrong , D. J. Casa , L. N. Belval , Nutr Res Rev 2019, 32, 205; 31258100 10.1017/S0954422419000076

[24] a) S. Borghini , S. Tassi , S. Chiesa , F. Caroli , S. Carta , R. Caorsi , M. Fiore , L. Delfino , D. Lasiglie , C. Ferraris , E. Traggiai , M. Di Duca , G. Santamaria , A. D'Osualdo , M. Tosca , A. Martini , I. Ceccherini , A. Rubartelli , M. Gattorno , Arthritis Rheum. 2011, 63, 830; 21360512 10.1002/art.30170 PMC3112487

[25] a) C. Ge , J. Hao , X. Wu , C. Li , R. Zhi , P. Yu , X. Wang , J. Hu , H. Xu , Lab. Anim. 2020, 54, 433; 31584316 10.1177/0023677219874844

[26] K. Y. Kwon , Y. J. Shin , J. H. Shin , C. Jeong , Y. H. Jung , B. Park , T. I. Kim , Adv. Healthcare Mater. 2019, 8, 1801593. 10.1002/adhm.201801593 31509350

[27] J. Yuan , R. Zhu , Appl. Energy 2020, 271, 115250.

[28] L. V. King , Proc. R. Soc. Lond. A 1914, 90, 563.

[29] S. Liu , J. Zhang , Y. Zhang , R. Zhu , Nat. Commun. 2020, 11, 5615. 33154381 10.1038/s41467-020-19424-2 PMC7645594

[30] P. Slade , M. J. Kochenderfer , S. L. Delp , S. H. Collins , Nat. Commun. 2021, 12, 4312. 34257310 10.1038/s41467-021-24173-x PMC8277831

[31] a) F. Shahid , A. Zameer , M. Muneeb , Chaos Solitons Fractals 2020, 140, 110212; 32839642 10.1016/j.chaos.2020.110212 PMC7437542

