# Supplementary material for: A Fully Self‐Powered Wearable Leg Movement Sensing System for Human Health Monitoring
Source: Adv Sci (Weinh). 2023 Aug 17;10(29):2303114. doi: 10.1002/advs.202303114 (PMC10582417; doi:10.1002/advs.202303114)
Supplement: Supplementary file 1 — Supporting Information [file ADVS-10-2303114-s001.pdf]

ADVANCED SCIENCE
Open Access

Supporting Information

for *Adv. Sci.*, DOI 10.1002/advs.202303114

A Fully Self-Powered Wearable Leg Movement Sensing System for Human Health Monitoring

*Jinfeng Yuan, Yuzhong Zhang, Caise Wei and Rong Zhu**

Supplementary Materials

A Fully Self-Powered Wearable Leg Movement Sensing System for Human Health Monitoring

Jinfeng Yuan *et al.*

*Corresponding author. Email: zr_gloria@mail.tsinghua.edu.cn

This file includes:

Supplementary Text
Figures. S1 to S7
Table S1 to S2
Note S1

Other Supplementary Materials for this manuscript include the following:
Movie S1

Table S1. Parameter definition

Parameter symbol	Physical meaning
V_1	General DC component of the output of f-TEG
V_2	General AC component of the output of f-TEG
ΔR	Resistance variation of strain sensor
R	Initial resistance of strain sensor
T_{air}	Ambient temperature
V_{s1}	DC component of the output of f-TEG on the shank
V_{s2}	AC component of the output of f-TEG on the shank
ΔT_s	Temperature difference between shank skin and environment
f_s	Gait frequency of shank
v	Motion speed
V_{t1}	DC component of the output of f-TEG on the thigh
V_{t2}	AC component of the output of f-TEG on the thigh
ΔT_t	Temperature difference between thigh skin and environment
f_t	Gait frequency of thigh
U	General output of f-TEG with constant temperature difference
I, J, m,n	General fitting coefficients
V	General output of f-TEG with temperature difference changes
ΔT	General temperature difference between skin and environment
f	General gait frequency
T_s	Shank skin temperature
T_t	Thigh skin temperature
I_{s1}, J_{s1}, m_{s1}	Fitting coefficients for the shank DC component
I_{s2}, J_{s2}, m_{s2}	Fitting coefficients for the shank AC component
I_{t1}, J_{t1}, m_{t1}	Fitting coefficients for the thigh DC component
I_{t2}, J_{t2}, m_{t2}	Fitting coefficients for the thigh AC component
θ	Knee joint angle
a	Vector sum of triaxial acceleration
a_x	X-axis acceleration
a_y	Y-axis acceleration
a_z	Z-axis acceleration

Table S2. Power consumption of each component in the system

(a) static mode

	ECM static mode			RTM static mode		
	Working current (I_w/μA)	Average working time per second (T_w/ms)	Equivalent power consumption (P_w/μW)	Working current (I_w/μA)	Average working time per second (T_w/ms)	Equivalent power consumption (P_w/μW)
MCU waits (in progress)	1.65	1000	3.63	1.65	1000	3.63
Accelerometer wake-up mode	2.05	1000	4.51	2.05	1000	4.51
Bluetooth remains connected				5.8	1000	5.8
Overall power consumption			8.1			13.9

(b) dynamic mode

	ECM dynamic mode			RTM dynamic mode		
	Working current (I_w/μA)	Average working time per second (T_w/ms)	Equivalent power consumption (P_w/μW)	Working current (I_w/μA)	Average working time per second (T_w/ms)	Equivalent power consumption (P_w/μW)
MCU waits (in progress)	1.65	1000	3.63	1.65	1000	3.63
MCU waits (preparation)	1800	0.5	1.98	1800	0.5	1.98
Accelerometer wake-up mode	2.05	1000	4.51	2.05	1000	4.51
Bluetooth remains connected				5.8	1000	5.8
SPI init	1800	1.05	4.158	1800	1.05	4.158
Getting accelerometer data	1800	1.15	4.554	1800	1.15	4.554
Signal conditioning circuit	4.12	0.65	0.006	4.12	0.65	0.006
ADC init and sampling	1900	0.9	3.762	1900	0.9	3.762
Data processing	1800	1.925	7.623	1800	1.925	7.623
Sending data through UART	2000	0.4	1.76	1800	5	19.8
Metabolic energy calculation	2000	5.815	25.586			
Speed/temperature calculation	2000	0.35	1.54			
Bluetooth broadcast mode	2000	7.5	33			
Bluetooth wakes up				2000	20.5	90.2
Bluetooth transparent mode				413	25	22.715
Overall power consumption			92.1			168.7

Each component can work independently both in hardware and in software. When testing a specific component, we provide power only to the corresponding component and run the corresponding program. The power supply voltage is 2.2 V. By monitoring the working current (I_w, achieved through dynamic signal acquisition (MPS-140801) and resistance voltage divider) and program execution time (T_w, achieved through oscilloscope)), we can calculate the equivalent power consumption (P_w) as follows:

$$P_w = I_w \times T_w / 1000 \times 2.2$$

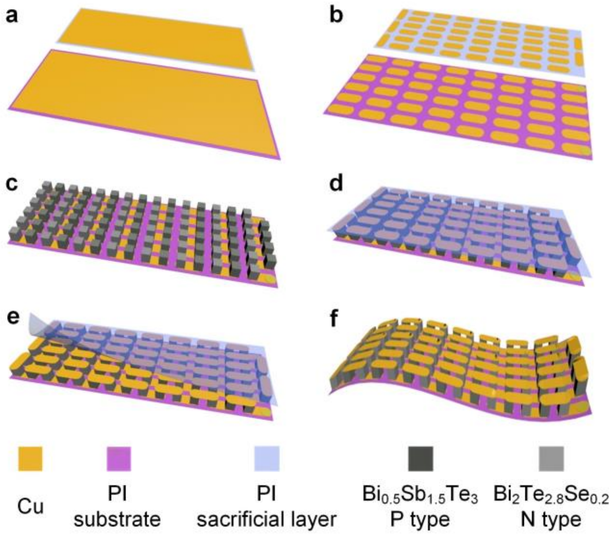

Figure S1: Fabrication process of f-TEG. a) Copper-coated PI film. b) Lift-off process. c) Hot welding particles. d) Coating sacrificial layer film. e) Acetone stripping. f) Final f-TEG.

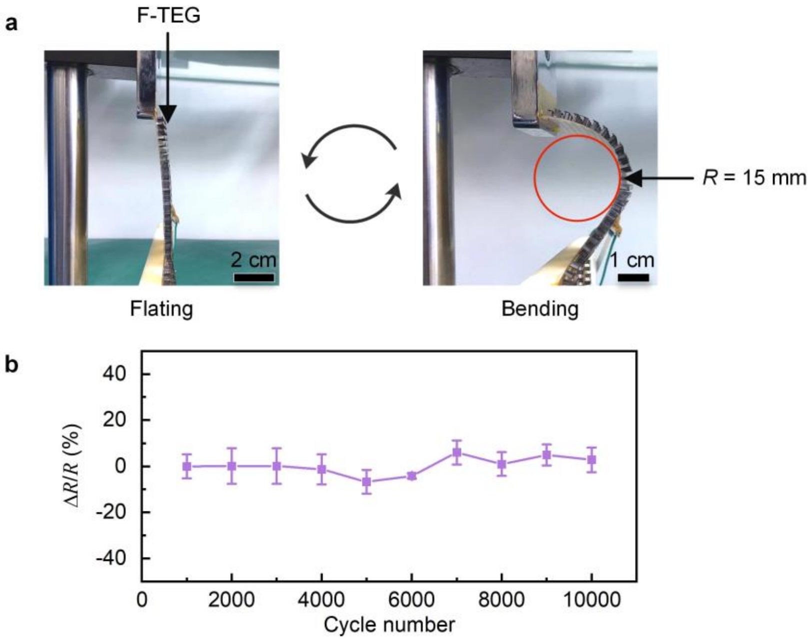

Figure S2. Durability test of the f-TEG under 10000 bending cycles. a) Experimental setup. The arc radius (R) of the f-TEG bending is 15 mm. b) Relative resistance change ($\Delta R/R$) of the f-TEG during 10000 bending cycles. The error bar is the standard deviation of three measurements. The results indicate that there is no significant damage to the f-TEG.

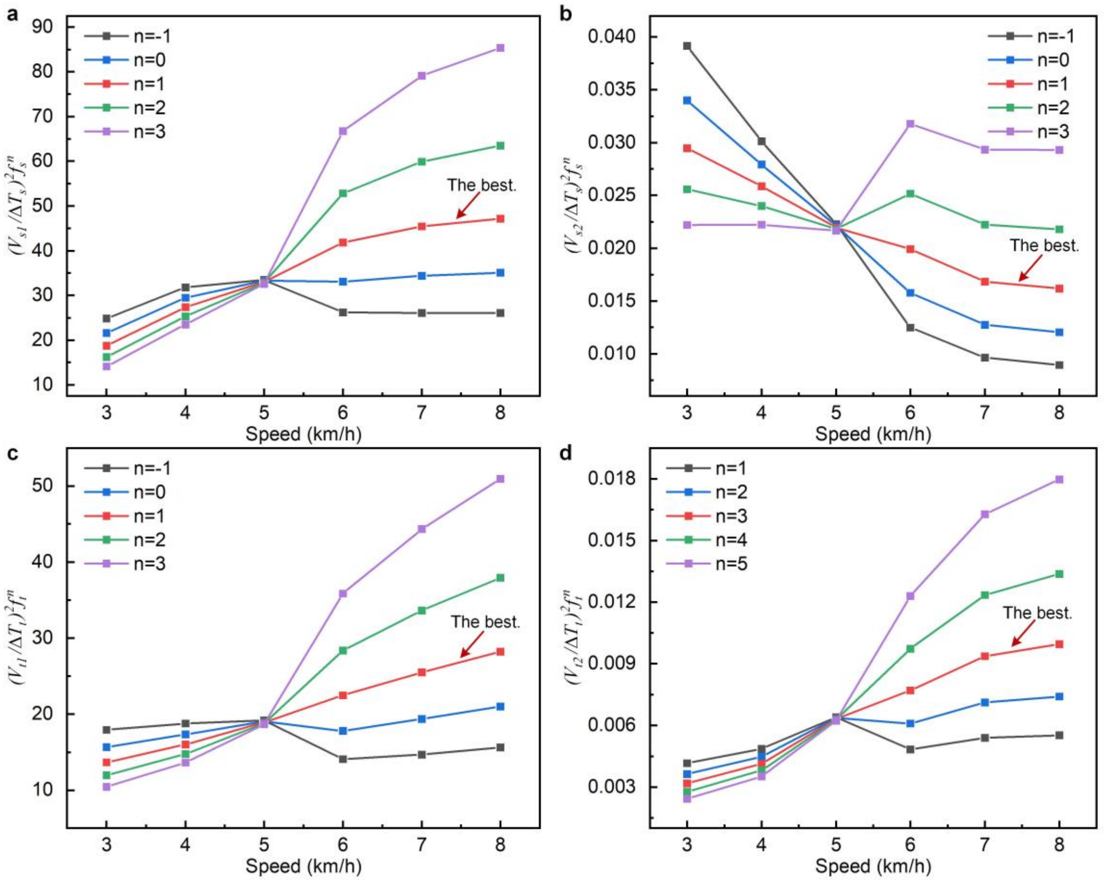

Figure S3. Value selection of *n*. Monotonic and smooth curve shape is beneficial to improve fitting accuracy for the general formula $(V/\Delta T)^2 f^{\,n} = I + J \times v^m$. The monotonicity and smoothness of the curve can be changed by changing the coefficient n. To reduce hardware computing complexity and power consumption, the definition field of n is set to an integer. a) Combined variable $(V_{s1}/\Delta T_s)^2 f_s^n$ vs motion speed v. Coefficient n=1 is considered the best choice. Other subsequent use cases follow the same rules. b) Combined variable $(V_{s2}/\Delta T_s)^2 f_s^n$ vs motion speed v. Coefficient n=1 is considered the best choice. c) Combined variable $(V_{t1}/\Delta T_t)^2 f_t^n$ vs motion speed v. Coefficient n=1 is considered the best choice. d) Combined variable $(V_{t2}/\Delta T_t)^2 f_t^n$ vs motion speed v. Coefficient n=3 is considered the best choice.

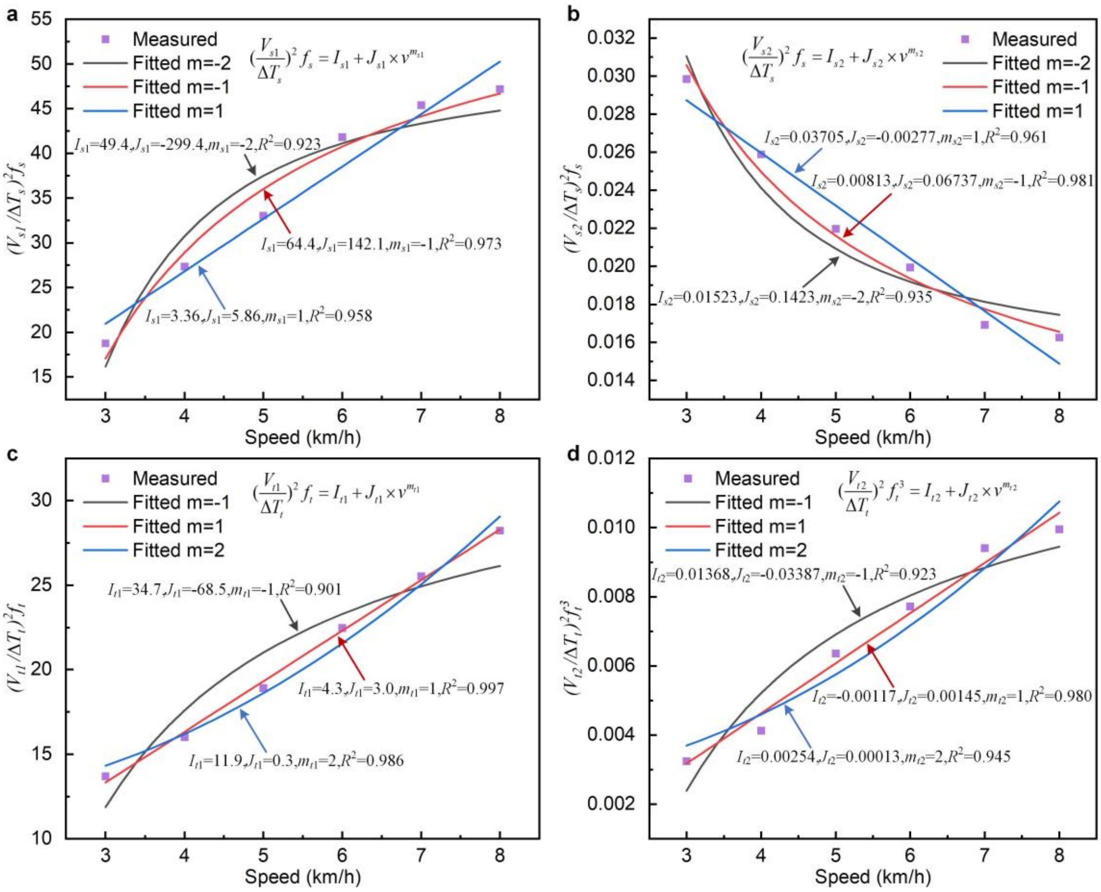

Figure S4. Value selection of *m*. To reduce hardware computing complexity and power consumption, the definition field of *m* is set to an integer. a) Influence of the value of *m* on the fitting of the combined variable $(V_{s1}/\Delta T_s)^2 f_s$ vs motion speed *v*. Coefficient *m*=-1 is considered the best choice. b) Influence of the value of *m* on the fitting of the combined variable $(V_{s2}/\Delta T_s)^2 f_s$ vs motion speed *v*. Coefficient *m*=-1 is considered the best choice. c) Influence of the value of *m* on the fitting of the combined variable $(V_{t1}/\Delta T_t)^2 f_t$ vs motion speed *v*. Coefficient *m*=1 is considered the best choice. d) Influence of the value of *m* on the fitting of the combined variable $(V_{t2}/\Delta T_t)^2 f_t^3$ vs motion speed *v*. Coefficient *m*=1 is considered the best choice.

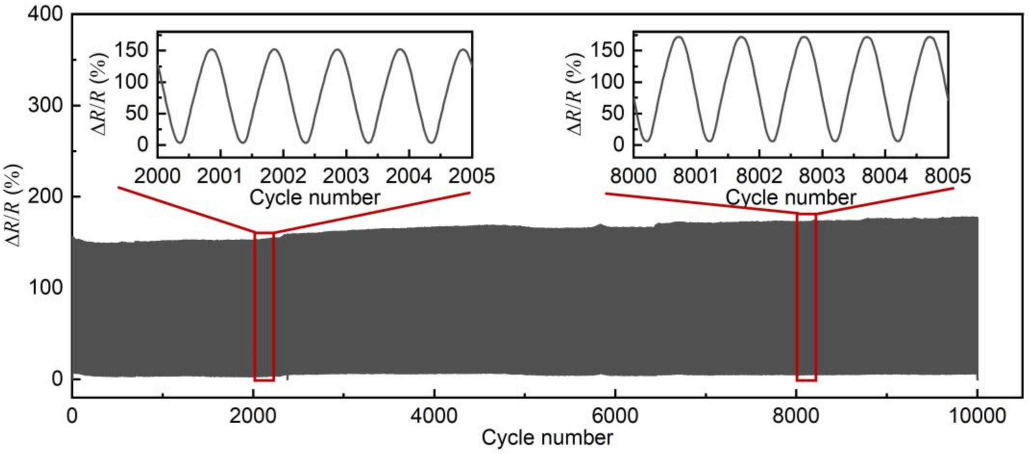

Figure S5. Fatigue resistance test of stretchable fabric strain sensor under 10000 stretching−releasing cycles. The cyclic strain is 20%. The result shows that the sensor has no obvious performance degradation.

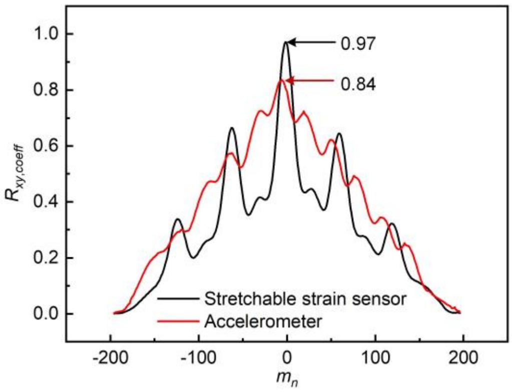

Figure S6. Normalized cross-correlation coefficient between the measured signal (the stretchable strain sensor or the accelerometer) and the actual signal (the knee joint angle measured by standard equipment).

The normalized cross-correlation coefficient $R_{xy,coeff}$ of two discrete time series $x(n)$ and $y(n)$ is calculated as follows:

$$R_{xy,coeff}(m_n) = \frac{R_{xy}(m_n)}{\sqrt{R_{xx}(0)R_{yy}(0)}} \tag{1}$$

Among them, $R_{xy}(mn)$, $R_{xx}(0)$, and $R_{yy}(0)$ are calculated by the following expressions:

$$R_{xy}(m_n) = \sum_{n=0}^{N-|m_n|-1} x(n)y(n+m_n) \tag{2}$$

$$R_{xx}(m_n) = \sum_{n=0}^{N-|m_n|-1} x(n)x(n+m_n) \tag{3}$$

$$R_{yy}(m_n) = \sum_{n=0}^{N-|m_n|-1} y(n)y(n+m_n) \tag{4}$$

where N is the total length of the sequence, m_n is the sequence index value, m_n=-(N-1), …, -1, 0, 1, …, N-1.

In this experiment, the time series dataset obtained in monitoring knee joint activity by the stretchable strain sensor or the accelerometer is $x(n)$, and the time series dataset obtained by the standard equipment is $y(n)$. It can be seen that the peak value of the normalized cross-correlation coefficient between the measured value of the strain sensor and the true value reaches 0.97, while the peak value of the accelerometer is only 0.84.

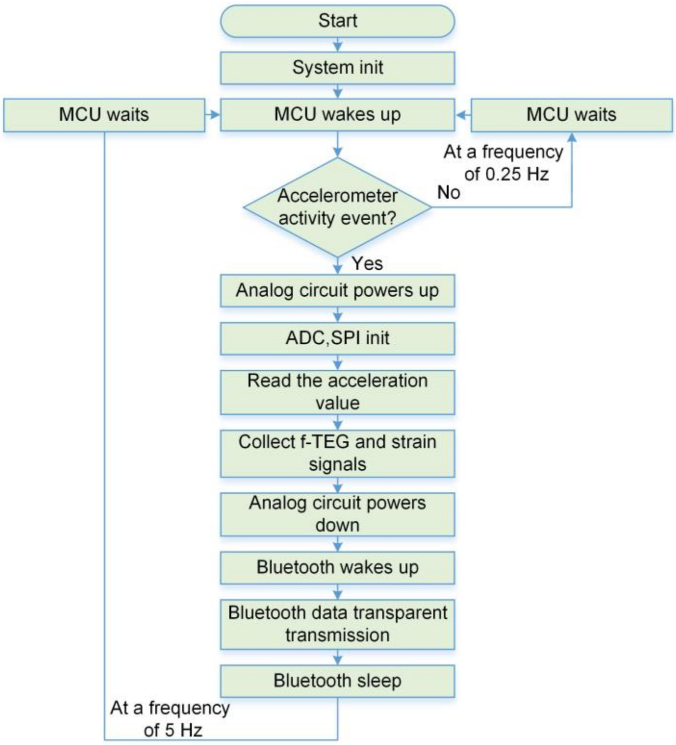

Figure S7. Workflow of RTM. The RTM uses the accelerometer signal to recognize whether the human body is active, and the MCU collects the accelerometer signal at a frequency of 0.25 Hz to reduce power consumption. In a quiescent state, the system enters a static mode in that the MCU does not collect the strain and f-TEG sensing data, and stops data transmission. Once a human activity is detected, the system enters a dynamic mode in that the MCU collects the strain sensing and f-TEG sensing data at a sampling rate of 5 Hz, and transmits the data to the external terminal through Bluetooth transparent mode.

Note S1. Calculation of motion speed and skin temperature of shank and thigh.
For shank parameters, there are formulas:

$$(\frac{V_{s1}}{\Delta T_s})^2 f_s = I_{s1} + J_{s1} \times v^{-1} \tag{5}$$

$$(\frac{V_{s2}}{\Delta T_s})^2 f_s = I_{s2} + J_{s2} \times v^{-1} \tag{6}$$

For thigh parameters, there are formulas:

$$(\frac{V_{t1}}{\Delta T_t})^2 f_t = I_{t1} + J_{t1} \times v \tag{7}$$

$$(\frac{V_{t2}}{\Delta T_t})^2 f_t^3 = I_{t2} + J_{t2} \times v \tag{8}$$

For shank skin temperature, thigh skin temperature and ambient temperature, there are formulas:

$$\Delta T_s = T_s - T_{air} \tag{9}$$

$$\Delta T_t = T_t - T_{air} \tag{10}$$

Combining the formulas 1 and 2, the motion speed *v* can be calculated as follow:

$$v = \frac{kJ_{s2} - J_{s1}}{I_{s1} - kI_{s2}} (k = (\frac{V_{s1}}{V_{s2}})^2) \tag{11}$$

Combining the formulas 1 and 5, the shank skin temperature T_s can be calculated as follows:

$$T_s = V_{s1}\sqrt{\frac{f_s}{I_{s1} + J_{s1}v^{-1}}} + T_{air} \tag{12}$$

Combining formulas 3 and 6, the thigh skin temperature T_t can be calculated as follows:

$$T_t = V_{t1}\sqrt{\frac{f_t}{I_{t1} + J_{t1}v}} + T_{air} \tag{13}$$
